# Hypoxia-adenosinergic regulation of B cell responses

**DOI:** 10.3389/fimmu.2024.1478506

**Published:** 2024-11-04

**Authors:** Layne Pruitt, Robert K. Abbott

**Affiliations:** Department of Pathology, University of Texas Medical Branch, Galveston, TX, United States

**Keywords:** hypoxia, B cell, antibody, adenosine, vaccine, metabolism

## Abstract

Hypoxic microenvironments induce widespread metabolic changes that have been shown to be critical in regulating innate and adaptive immune responses. Hypoxia-induced changes include the generation of extracellular adenosine followed by subsequent signaling through adenosine receptors on immune cells. This evolutionarily conserved “hypoxia-adenosinergic” pathway of hypoxia → extracellular adenosine → adenosine receptor signaling has been shown to be critical in limiting and redirecting T cell responses including in tumor microenvironments and the gut mucosa. However, the question of whether hypoxic microenvironments are involved in the development of B cell responses has remained unexplored until recently. The discovery that germinal centers (GC), the anatomic site in which B cells undergo secondary diversification and affinity maturation, develop a hypoxic microenvironment has sparked new interest in how this evolutionarily conserved pathway affects antibody responses. In this review we will summarize what is known about hypoxia-adenosinergic microenvironments in lymphocyte development and ongoing immune responses. Specific focus will be placed on new developments regarding the role of the hypoxia-adenosinergic pathway in regulating GC development and humoral immunity.

## Introduction

Hypoxic microenvironments routinely exist in tissues under normal physiological settings but also can develop in response to infection, inflammation, tissue injury or neoplastic growth. These microenvironments limit or redirect immune responses to protect tissues from excessive collateral damage caused by inflammation. It has been proposed that hypoxia may be one of the oldest evolutionarily conserved immunoregulatory pathways.

**Figure 1:**
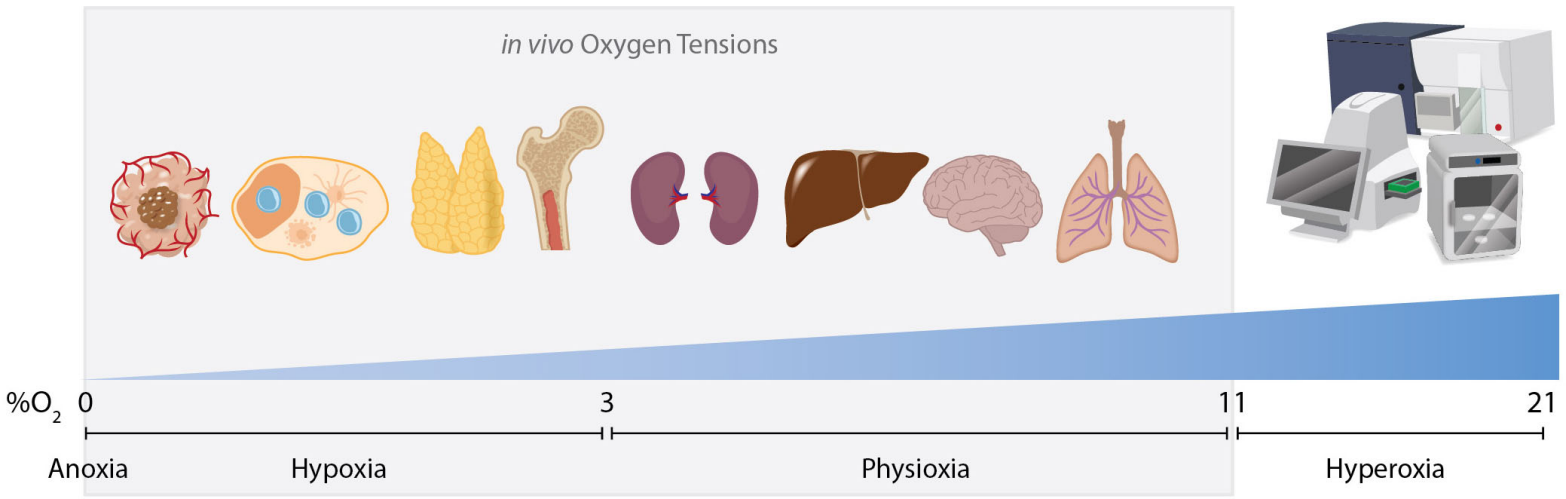
Oxygen tensions *in vivo* and *in vitro.* Cells are exposed to various oxygen tensions within the body and in culture conditions. Hypoxic microenvironments exist routinely within the body in various tissues such as lymph nodes, the thymus and bone marrow. Hypoxia also commonly occurs in pathologies such as solid-tumor malignancies or areas of local inflammation. Most organs exist in a state of physioxia, an oxygen tension that is normal for that tissue but is not low enough to stabilize Hifs, ranging from 3% to 11% O_2_. *In vitro* cells are typically exposed to a supraphysiological concentration of oxygen as ambient air in incubators/sorters/seahorse analyzers and other standard instruments is ~21% O_2_. High speed cell sorters increase dissolved oxygen concentrations even further as high pressure is used to achieve high sort speeds. This may complicate interpretations when analyzing cells whose physiological niche is hypoxic.

Hypoxia (low oxygen tensions) stabilizes proteins that are master regulators of the cellular response to hypoxia. These so-called hypoxia inducible factors (Hifs) (1) primarily consists of 3 known isoforms; Hif-1α, Hif-2α, and Hif-3α. Hifs are generally stabilized when the concentration of oxygen drops below 3% O_2_ (e.g. ~22mmHg paO_2_) (2). While normal air consists of 21% O_2_ (“normoxia”), the highest concentration of oxygen *in vivo* occurs in the lung, typically between 7 and 11% O_2_ (3) (**Figure 1**). Moreover, many tissues (including lymphoid tissues such as the spleen and lymph nodes) exhibit even lower oxygen concentrations (0.5-5% O_2_) (4) than levels detected in the blood. In general, oxygen tensions are categorized as anoxic (0% O_2_), hypoxic (2) (0%→3%), physioxic (5) (3→11% O_2_), and hyperoxic (11%→100% O_2_) (**Figure 1**). It is worth noting that most of the widely used, standard cell culture incubators utilize 21% O_2_ and thus fall in a supraphysiological hyperoxic category. A second technological point is that hypoxic microenvironments are often detected using nitro-imidizole based reagents. These compounds are reduced in hypoxic conditions and selectively bind to proteins under extremely low oxygen tensions (<~1.2% O_2_) (6). In addition, direct immunostaining of Hif-1α is often a formidable challenge due to the extraordinarily short half-life (less than 5 minutes in normoxic air) (7). Thus, it is likely due to the well-known limitation of detection methods that many hypoxic microenvironments (1.2% O_2_ →3% O_2_) remain underreported.

**Figure 2:**
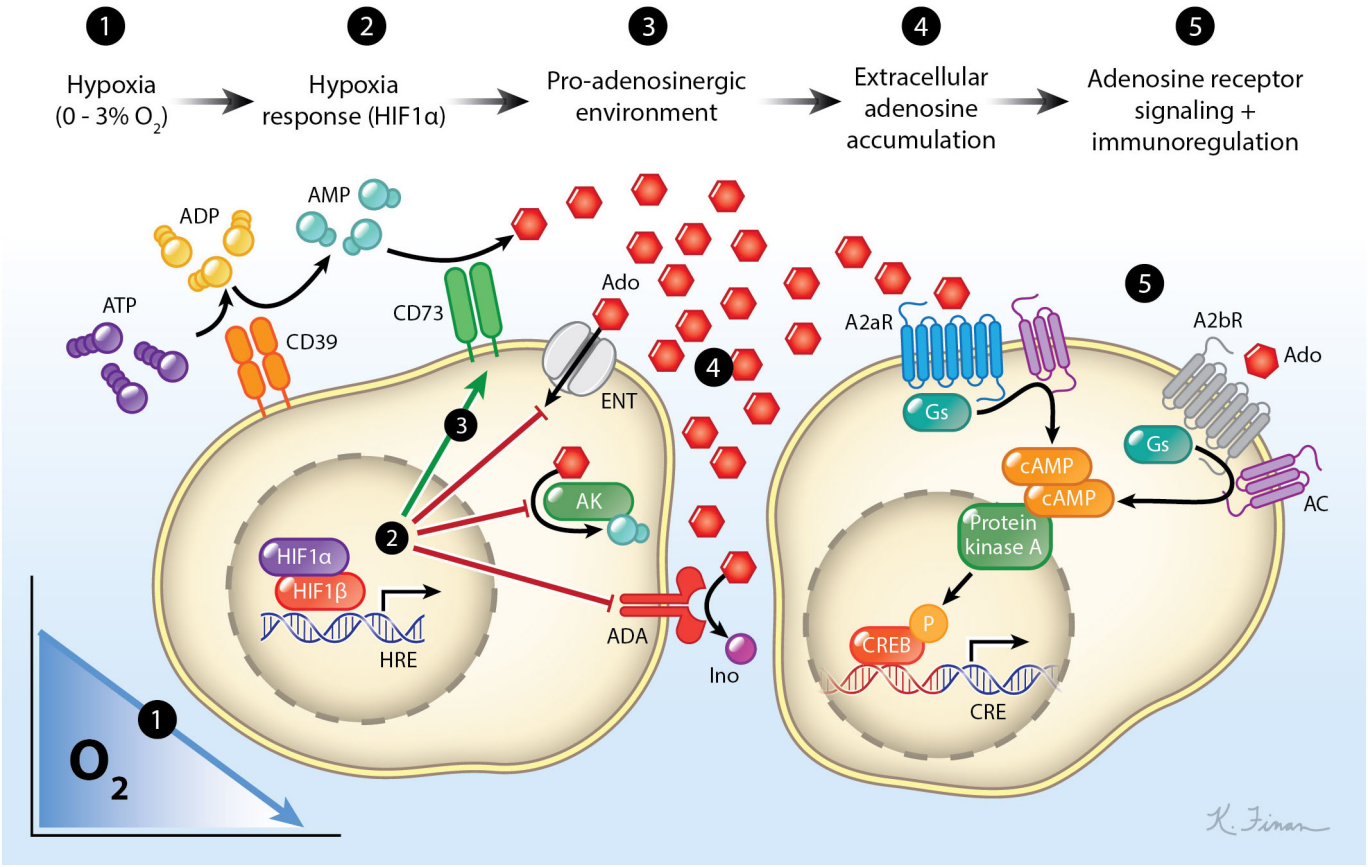
The hypoxia-adenosinergic pathway. As cells experience hypoxia, they stabilize the transcription factor Hif-1α which induces the expression of HREs. These HREs include enzymes that catabolize extracellular ATP to adenosine (CD39/CD73), prevents the movement of extracellular nucleosides into the cell, prevents phosphorylation of nucleosides, and prevents the catabolism of adenosine to inosine. This leads to an accumulation of extracellular adenosine which can then signal through adenosine receptors. The most abundant adenosine receptors expressed on lymphocytes are A2aR and A2bR which both can couple to the Gs protein subunit. Signaling through these receptors activates adenylyl cyclase leading to an accumulation of cAMP within the cell. This activates the protein kinase A to phosphorylate the cAMP responsive element binding protein (CREB), which leads to immunomodulation. For simplicity, A1 and A3 adenosine receptors and their pathways are not shown.

Hypoxic microenvironments often initiate a cascade of events that leads to a net increase in the concentration of extracellular adenosine (eAdo) (8). This eAdo subsequently signals through four known adenosine receptors (A1R, A2aR, A2bR, and A3R) to modulate immune responses (9, 10). This signaling axis (hypoxia → Hif stabilization → increase in eAdo generating enzymes [e.g. CD73, CD39, etc.] → adenosine receptors → immunomodulation) is collectively referred to as the “hypoxia-adenosinergic” pathway (11) (**Figure 2**). The hypoxia-adenosinergic pathway has been implicated in governing immune suppression in a various disorders including infectious disease (12), autoimmunity (13), cancer (14), inflammation (10), and most recently in the germinal center (GC) reaction (15–21). The discovery of the important role of this pathway in the GC reaction may be crucial since this is where B cells undergo secondary diversification and the process of affinity maturation (AM) to elicit broadly neutralizing anti-pathogen antibodies.

In this review we will give a brief overview of how hypoxia, eAdo signaling, and metabolic signaling affects lymphocytes with a special emphasis on B cells. We will also summarize how hypoxia influences both development and effector functions of lymphocytes. We will pay particular attention to the recent discovery that the GC is hypoxic and therefore is under the control of the hypoxia-adenosinergic pathway.

## Tissue hypoxia drives a pro-adenosinergic microenvironment

The cellular response to hypoxic conditions is facilitated by Hif proteins that rapidly respond to changes in oxygen tensions. This pathway consists of a cytoplasmic surveyor for hypoxia [Hif-1α (22)/Hif-2α (23–25)/Hif-3α (26)] and a nuclear responder (Hif-1β (22)). Hif-1α is widely expressed in immune cells (27). Hif-2α is more restricted in expression which is found in leukocytes like macrophages as well as tumor associated T lymphocytes (28). This protein complex is regulated principally at the protein level rather than transcriptionally (29). The alpha subunits are targeted by the prolyl hydroxylase domain (PHD) proteins, which hydroxylate proline residues [P402 and/or P564 for Hif-1α (30), P405 and/or P531 for Hif-2α (31)] in physioxic conditions. This hydroxylation facilitates binding of an E3 ubiquitin ligase called the von Hippel-Lindau protein (pVHL) (32). pVHL ubiquitination orchestrates continual proteasomal degradation of Hif-1α (33). When oxygen levels drop to hypoxic levels the ability of the PHD enzymes to hydroxylate the Hif-α subunits is impaired resulting in immediate stabilization of Hif-1α. While this is the principal method for Hif regulation, it should be noted that there are non-canonical methods to stabilize Hifs (34).

Upon stabilization, Hif-1α translocates to the nucleus of the cell to dimerize with the constitutively expressed Hif-1β subunit to form a mature Hif dimer (22). Hif dimers then bind to specific sequences in the genome called hypoxic response elements (HREs). Hif binding to these HREs has been demonstrated to have an enhancer type function that can successfully recruit other transcription factors, nuclear proteins, and direct DNA binding proteins to cooperatively enhance RNA polymerase function at these sites (35, 36). While the targets of Hif signaling vary dramatically between different cell types, there are multiple conserved pathways that allow adaptation of cells to stressful environmental conditions. These HRE induced pathways promote oxygen delivery and reduce oxygen consumption/demand (37). This reduction of oxygen demand comes from inhibiting oxidative phosphorylation (OxPhos), the primary method by which most cells create ATP. To compensate for this loss in energy demand, Hifs mediate an increase in in glucose transporters (GLUTs) and glycolytic enzymes (38, 39). Hifs also upregulate signals to improve oxygen delivery to hypoxic tissues such as vascular endothelial growth factor (40, 41), erythropoietin (42) and transforming growth factors (43, 44).

Hypoxia alters the concentration of extracellular ATP (45–47). In normal physiological tissue, ATP is found at substantially higher concentrations intracellularly rather than extracellularly. A typical cell contains 5 – 10 mM of ATP intracellularly (48). Conversely, extracellular concentrations of ATP are rather sparse and estimated to be in the nanomolar range (9, 49). Hypoxia induces a multitude of changes that can lead to release of intracellular ATP, such as autophagy (50), release of ATP bearing vesicles (51), and extrusion through cellular channels (9). Autophagy, the process by which intracellular components are degraded and recycled, has been shown to be promoted by hypoxia (52). Multiple reports demonstrate that autophagy is directly linked to an increase in the secretion of damage associated molecular patterns (including ATP) (53–56). Recent advances in the ability to isolate and characterize extracellular vesicles (EVs) has suggested a role in hypoxic pathophysiology. Though it is difficult to ascertain if these are true EVs or artifacts of tissue processing techniques creating fragmented cellular debris. One emerging finding is that these small vesicles can hold ATP (57), and the production of EVs is increased under hypoxic stress (58). Finally, there are several receptors and channels that exist on the plasma membrane that have been found to pump ATP out of the cells in pathological conditions. Connexin (59), pannexin (60), and CALHM1 (61) channels have been suggested to mediate ATP release from a cell. These channels and more have been thoroughly reviewed by the Virgilio group (9). All these factors contribute the role of hypoxia in orchestrating a dramatic increase in the levels of extracellular ATP.

Hypoxic microenvironments promote catabolism of extracellular ATP. Cells in hypoxia upregulate the expression of nucleotidase enzymes of their surface that catabolize ATP in the extracellular space to the nucleoside adenosine. While other adenosine-generating pathways exist, much attention has focused on the tandem activity of the ectoenzymes CD39 (62–64) and CD73 (62, 63, 65–67). CD39 is responsible for converting extracellular ATP to either ADP or AMP (68) while CD73 (69) catabolizes AMP to adenosine. These ectoenzymes are not ubiquitously expressed but are found in lymphocyte subsets in specific conditions such as hypoxia or upon activation (15, 70–72). This suggests that lymphocytes in hypoxic conditions are predisposed to both create and encounter eAdo, which also might indicate a possible autocrine signaling loop as eAdo can then signal through adenosine receptors. All four adenosine receptors are g coupled proteins (73), but the receptors pre-dominantly expressed by the adaptive immune system are A2aR and A2bR (11, 74). A3R has also been found on human B cells and leukocytes such as mast cells (75–77). A2aR and A2bR couple with the g_s_ subunit when signaled through, activating adenylyl cyclase and increases the concentration of cAMP inside the cells (78). Notably, A2bR has long been considered the “low affinity” adenosine receptor due to its affinity for 5’-N-Ethylcarboxamidoadensinebeing (NECA) being ~100-fold lower than that of A2aR (79).

The first evidence for an *in vivo* role of eAdo attenuating inflammation through adenosine receptors was a landmark study by Ohta and Sitkovsky in 2001. Upon injection of sub-maximal inflammatory stimuli, the authors demonstrated that mice genetically lacking A2aR had an exacerbated immune response. The authors principally used a concanavalin-A induced liver injury model, but also utilized pseudomonas aeruginosa endotoxin A, carbon tetrachloride, and lipopolysaccharide induced inflammation models. In response to inflammation, A2aR KO mice had excessive tissue damage, higher and more prolonged pro-inflammatory cytokine response, and even death of some male mice when compared to littermate controls (80). This finding established the hypoxia-adenosinergic pathway as a prominent pathway in limiting and redirecting inflammation *in vivo*.

In sum, hypoxia induces changes to the microenvironment that influences surrounding cells. Hypoxia causes a sharp increase in the concentration of extracellular metabolites such as extracellular ATP. Cells experiencing hypoxia also upregulate the expression of ectoenzymes that can catabolize extracellular ATP to eAdo which serves as a signaling ligand for adenosine receptors which are also upregulated by hypoxia. Altogether this cascade has been coined the hypoxia-adenosinergic pathway.

## Role for hypoxia and metabolic signaling in B cell development

Early direct genetic evidence that hypoxia plays a substantial role in B lymphocyte development came from seminal studies from the Sitkovsky group (81) by ablation of Hif-1α in the lymphocyte compartment by utilization of the elegant Rag 2 blastocyst complementation system (82). Germline deletion of Hif-1α results in embryonic lethality by day 14 (83). One method to circumvent this problem is to inject Hif-1α-deficient embryonic stem cells into a Rag2-KO blastocyst. This results in both preventing embryonic lethality and producing chimeric progeny in which only lymphocytes genetically lack Hif-1α (with germline efficiency) (63). In these mice, the largest perturbations observed were specifically in the B1 cell compartment in the peritoneal cavity, as well as alterations in the bone marrow in specific stages of B cell development (81). Surprisingly, there were no gross defects in CD4 or CD8 T cell populations or any noted in the peripheral B2 cell compartment in naïve mice. This may be due to the fact that the peritoneal cavity is potentially a constitutively hypoxic region (direct measurements as low as 17 mmHg O_2_ have been observed in the peritoneal cavity, corresponding to ~2.2% O_2_) (84) and thus major defects are more observable at baseline as B1 cells are chronically exposed to hypoxia. One of the most curious and interesting finding from this work was that genetic ablation of Hif-1α in lymphocytes had a dramatic effect on autoreactive antibodies. In Hif-1α^-/-^ chimeric mice there was an increase in the levels of serum anti-dsDNA IgG and IgM as well as substantial IgM and IgG deposits in the kidneys, proteinuria, and the accumulation of rheumatoid factor (81). While there was a net increase in IgM, as well as more observed perturbations in the B1 cell compartment, it is unknown if the break in tolerance in these mice is B1 or B2 cell derived and if any of these autoantibodies are germinal center (GC) derived.

Subsequent study revealed that this Hif-1α deficiency in B cell development led to dramatic reductions in key molecules involved in Hif-1α mediated glycolysis (85). Namely, there were specific reductions in GLUTs 1 and 3, as well as the key glycolytic enzyme 6-phosphofructo-2-kinase/fructose-2,6-bisphosphatase 3 (85). Interestingly, *in vitro* bone marrow cultures revealed that Hif-1α^-/-^ B cells compensated for the loss of glycolytic capacity by upregulating the capacity to utilize pyruvate as an energy source in the absence of glucose.

Overexpression of Hifs also leads to significant perturbations in the B cell development. This was achieved by conditional knock-out of the pVHL gene. Without continual degradation by pVHL, Hifs are stabilized and accumulate in cells under hypoxic and non-hypoxic conditions. This perpetual stabilization leads to potential over-accumulation of complete Hif dimers which is not observed physiologically in hypoxic tissues and may impact interpretations. Constitutive Hif stabilization by deletion of the pVHL gene leads to a loss of B cell subsets as well as reduced Vh utilization and clonal diversity (86). In pVHL-deficient B cells there is also a downregulation of BCR signaling pathways, an increase in restrictive gene expression (FOXO1, BCL2L11), and a developmental blockage at the immature B cell stage (86). Deletion of the gene controlling apoptosis in B cell development, BIM, ameliorated the developmental stall and restored peripheral mature B cell populations (86). To explore the mechanism of this, Mb1-cre VHL^fl/fl^ mice were bred to either Hif-1α^fl/fl^ or Epas1^fl/fl^ (Hif-2α) mice to delete desired genes. Hif-1α was found to be the major driver of this phenotype, suggesting a more prominent role for Hif-1α in B cell development (86). Transcriptomic analysis from these Vhl-deficient immature B cells suggested that this stagnation in development was related to uncontrolled Hif-1α reducing BCR signaling and hindering survival. Interestingly, pVHL deficient mice (by a Vhl^fl/fl^ CD19-cre system) were demonstrated to have a reduction in mature naïve B cells (87). This was attributed to improper decoupling of glycolysis from the tri-carboxylic acid cycle (TCA). Mechanistically, dysregulation of this pathway leads to improper utilization of imported carbons for fatty acid oxidation. This leads to palmitoylation of the FAS receptor (87), resulting in increased death of mature B cells in a caspase 8-dependent manner. This is particularly interesting as it demonstrates that when B cells have an improper metabolic paradigm in place under physiological hypoxia, complications appear to be more nuanced than simply not meeting energetic demands.

All lymphocytes arise from hematopoietic stem cells (HSCs) that reside in the bone marrow (BM). B cell specific lymphocyte differentiation is committed following Pax5 (88) and Ebf1 (89) expression (90). After B cell lineage commitment, cells enter a pre-pro B cells stage (Hardy Fraction A) which undergo diversity (D) and junction (J) [(D)J] rearrangement of their immunoglobulin heavy chain. Pre-pro B cells are developed in the sinusoids of the BM (91). HSCs are also housed in this sinusoid space within the BM (92, 93), a region known to be poorly vascularized (94). Pre-pro B cells have the highest staining of pimonidazole (PIMO) of the Hardy Fractions (95), suggesting they develop in intensely hypoxic regions of the BM.

After successful (D)J rearrangement in the BM, pre-pro B cells transition into pro-B cells (Hardy fraction B-C). Chicana et al. showed that pro-B cells show lesser PIMO staining than pre-pro B cells (95), but are still positive for PIMO staining. Positive PIMO staining suggests that these cells are still within a hypoxic microenvironment. Notably, it was observed as B cells progressively developed, each subsequent stage appeared to have diminished PIMO staining. This suggests that as B cells develop in the bone marrow, they may progressively migrate toward more oxygenated environments. At the pro-B cell stage in development, RAG genes are expressed, and B cells undergo heavy-chain rearrangement of their variable (V) regions and adjoin them to the rearranged (D)J segment. Pro-B cells demonstrate a dependence on reactivity to IL-7 (96, 97) that signals migration and localization to stromal cells that express IL-7 (98). Upregulation of the IL-7 receptor on developing B cells is required for proper chemotaxis (99) from the sinusoids. Curiously, a downregulation of IL-7 was noted in hypoxic environments has been reported in several studies (100, 101). In terms of B cell development, this may be in line with the hypothesis that when pro-B cells migrate into slightly more oxygenated environments, they upregulate the means to interact with IL-7 which is required for further differentiation (100, 101). Alternatively, pro-B cells could still be in hypoxic enough niches to squelch IL-7 expression, encouraging a competitive environment for developing B cells to respond to stringently regulated IL-7 levels. This later hypothesis draws parallels to GC reactions in which clonal bursts of GC B cells is evident following productive interactions with Tfh, even though Tfh are known to be “stingy” producers of helping cytokines such as IL-4 and Il-21 (102). IL-7 is known to induce a significant proliferative burst in pro-B cells (89, 103, 104). This expansion of pro-B cells was shown to be reliant on glycolysis and Hif-1α expression (85). Defects in glucose transporters such as Glut1 and Glut3 or glycolytic enzymes like phosphoglycerate kinase 1 stop development of B cells at this pro-B cell stage (85). Hypoxia appears to be indispensable for the proper development of B cells with a significant stage specific impact particularly at the pro-B cell stage.

Pre-B cells (Hardy fraction C-D) are the next developmental stage. These cells display less PIMO staining compared to Pro-B cells (95), but are still positive for PIMO staining. Globally, this suggests pre-B cells may reside in a more oxygenated environment. Pre-B cells first emerge as large pre-B cells, so named because they substantially increase both their biomass and metabolic machinery to accommodate a proliferative burst. Metabolically, large pre-B cells display a significant increase in both glucose uptake and OxPhos, leading to an increase in lactate production and reactive oxygen species (ROS) production by these cells (105). This metabolic increase could be attributed to pre-BCR signaling downregulating EFhd1 (106), which negatively regulates mitochondrial respiration and glycolysis. As large pre-B cells divide, they begin to lose biomass. Subsequently, signaling through the pre-BCR complex appears to diminish responsiveness to IL-7 while upregulating FOXO1 (107). FOXO1 restriction of glycolysis and OxPhos genes facilitates the transition of developing B cells into the small pre-B cell stage (107). Small pre-B cells enter a state of quiescence which allows time to undergo VJ rearrangement of their light chain genes to produce a mature BCR (106). Rictor, the downstream complex from mammalian target of rapamycin complex (mTORC) 2 signaling, was found to be vital at this stage (108). Rictor was found to be the driver of the significant expansion of pre-B cells in the BM (108). Another critical regulator of B cell development is the Fnip1 gene. Fnip1 canonically regulates the AMPK pathway which represses mTORC activation (109). There is an increase in cell size and an elevated level of cellular death due to the dysregulation of the mTORC/AMPK pathways when Fnip1 is deleted (110, 111). After a light chain is successfully synthesized and a complete BCR is expressed (and not selected against in the bone marrow) these cells exit the bone marrow and migrate to the spleen as immature B cells.

Immature B2 cells (Hardy Fraction E) enter the spleen to complete development. As immature B cells enter the spleen, they become transitional B cells. Transitional B cells then begin differentiating into either a follicular (FO) B cell or a marginal zone (MZ) B cell in mice. FO B cells undergo another metabolic shift to become quiescent sentinels of secondary lymphoid organs. FO B cells downregulate a myriad of anabolic and proliferation associated genes involved with protein synthesis, aerobic respiration, and mTORC signaling. In agreement with this, FO B cells display a lower basal oxygen consumption rate (OCR) and extracellular acidification rate (ECAR) when compared to transitional B cells (112). OCR is a measurement of how much oxygen is being consumed by OxPhos while ECAR is a measurement of how much lactic acid is being produced by cells as a result of glycolysis. The diminishment of both readouts demonstrates that FO B cells substantially downregulate their metabolic activity. Human FO B cells also increase expression of CD39 and CD73, thereby increasing adenosine production and inducing activation of the AMPK pathway which is a known silencer of mTORC1 preventing cell division (112).

**Figure 3:**
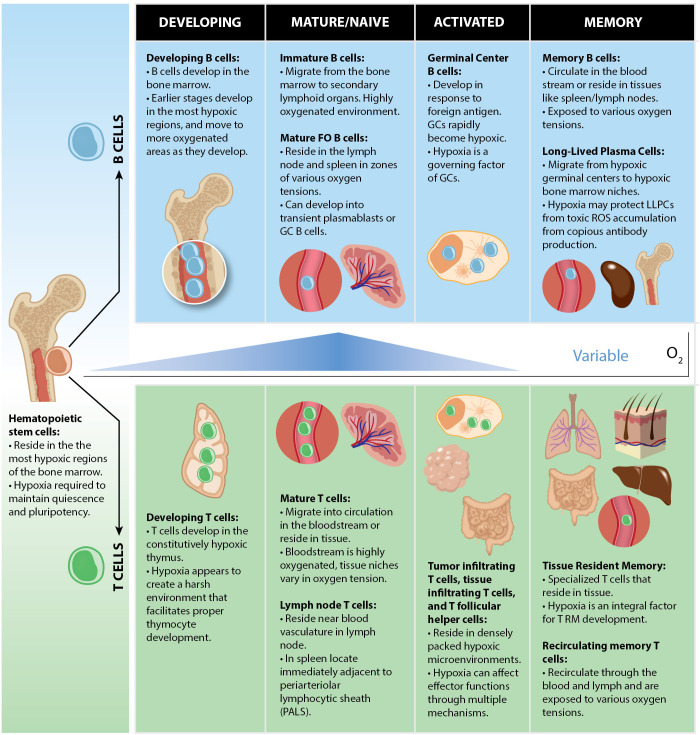
Oxygen tensions throughout B and T cell lymphocyte development. Both B and T cells arise from HSCs which are localized within the most hypoxic regions of the BM. Commitment to the lymphoid lineage differentiates HSCs to the common lymphoid progenitor (CLP) stage, still within with hypoxic bone marrow niches. B and T cells each develop in hypoxic microenvironments in which V-D-J recombination occurs (BM for B cells, thymus for T cells). When they mature, they can either circulate in the bloodstream or reside in tissues in physioxic microenvironments ranging from 3 to 11% O_2_. Activated lymphocytes encounter hypoxia following activation under certain conditions such as in GCs, tumors, or gut mucosae which may serve to promote or restrain their effector functions.

In sum, B cells are exposed to a myriad of microenvironments and varying oxygen tensions during B cell development in the bone marrow (**Figure 3**). Proper development of B cells requires the appropriate metabolic programs to be employed to proceed to the next checkpoint. Hypoxia, through Hifs (particularly Hif-1α) plays a vital role in this process as Hif-mediated metabolic adaptations are instrumental in the survival within the hypoxic bone marrow. This is evident as both overexpression, and genetic removal of Hif-1α in B cells, lead to significant defects in B cell development and central tolerance.

## Role for hypoxia in regulating germinal center responses

GCs are microanatomical structures located in secondary lymphoid organs (SLOs) where B cells undergo iterative selection events (113) to develop high affinity antibodies. GC B cells undergo somatic hypermutation (SHM) of their antibody genes to gain affinity to their cognate antigen/epitope. SHM is governed by the enzyme activation induced cytidine deaminase (AID) (114, 115). AID induces mutations directly in the immunoglobulin locus (116). Following recognition of specific DNA sequence “hotspots” AID deaminates cytidine nucleotides to uracil (114). These mutation events are thought to occur in the dark zone (DZ) of the GC. DZ GC B cells undergo substantial proliferative bursts while experiencing SHM (113), then migrate to the light zone (LZ) of the GC. The LZ is where GC B cells undergo Tfh mediated selection (117). If the mutation is beneficial for antigen binding, the subsequent clones are generally positively selected. During this selection in the GC, B cells with mutated BCRs compete for antigen retained on follicular dendritic cells, internalize, then present the antigen to cognate Tfh to receive survival stimuli. GC B cells that receive enough Tfh cell-help avoid apoptosis and undergo one of three fates: exit from the GC as either a memory B cell (MBC) (118) or long-lived plasma cells (LLPCs) (119), or movement back into the DZ to expand, mutate, and compete for selection again (117). This process has been coined the ‘cyclic re-entry’ model of GC selection (120) and explains the stepwise nature of SHM accrual and antibody affinity. Negative selection in GCs is thought to resolve principally by apoptosis due to insufficient T cell help.

Collectively, the mutation and selection process within GCs mechanistically explains the phenomenon known as affinity maturation (121). Affinity maturation is characterized by the net improvement (upwards of 10,000-fold in K_D_) of serum antibody affinity in the months following vaccination (122). B cells that have undergone affinity maturation can leave the GC as either MBCs or LLPCs. LLPCs migrate to specialized bone marrow niches to consistently secrete thousands of immunoglobulin molecules per second and can last a lifetime (123, 124). MBCs can either recirculate or become tissue resident MBCs (125, 126). Importantly, several recent studies have demonstrated that the GC develops a hypoxic microenvironment that is critical for the GC reaction (127–129).

Antibody and complement opsonization of antigen have been demonstrated to contribute to GC success (130, 131). Early antibody, at least partially, arises from plasmablasts. Plasmablast activation is typically early after antigen exposure and the result extrafollicular B cell activation which can provide a level of acute antibody for a short duration (a couple of days to weeks) (119). Activation of B cells leads to class switch recombination (CSR) where chromosomal loci are exchanged within B cell genomes to alter expression of the constant genes that define antibody isotype (132). Like SHM, this process is governed by AID. Naïve B cells express IgM/IgD immunoglobulins on their surface. CSR facilitates expression of other isotypes such as IgG, IgE, and IgA (133). These different isotypes have different immunological properties (134). Switching to each of these classes is dictated by specific cytokine signals (135).

Recently, hypoxia has been shown to enhance the ability of activated B cells to become antibody secreting cells (ASCs) (127, 136). We hypothesized that GCs may be hypoxic and potentially outgrow the vascular bed as they rapidly form in the days that follow vaccination or infection. Indeed, they found that GCs robustly bind PIMO, indicating the GC is predominantly a hypoxic microenvironment. To functionally assess the role of hypoxia on B cells *in vitro*, they found that when murine B cells are stimulated to undergo CSR in hypoxic conditions (1% O_2_) under IgG1 polarizing conditions, there is an acceleration of B cell proliferation and CSR kinetics. Concomitant with this was an enhanced generation of B220^-^CD138^+^ plasma cells under hypoxic culture conditions (127). Curiously, after 4 days of hypoxic cell culture, B cells showed substantial increase in the apoptotic marker active-caspase 3. As apoptosis is a key hallmark of GC physiology (137), hypoxia may play a key role in “setting the stage” for normal GC function. Moreover, activation of B cells under hypoxic cell culture conditions leads to dramatic upregulation of the prototypic GC marker GL7 (127). *In vitro* studies of human B cell activation in hypoxic conditions by the Rispen group show similar findings, suggesting the B cell response to hypoxia is phylogenetically conserved. When human B cells are activated in hypoxic culture conditions a unique subset of B cells that express high levels of CD27 develop (138). This CD27 high population was found to be quiescent after development. However, following restimulation *in vitro*, this population had the capacity to rapidly differentiate into ASCs (138). While low oxygen tension seems beneficial for the GC and ASC performance in their specific niches, systemic hypoxia appears to be detrimental to humoral responses. The Smith group analyzed B cell responses in a cohort of patients with COVID-19 at various severities. Systemic hypoxia appeared to reduce IgM titers in the serum of these patients. Reduced SHM in class switched B cells and altered utilization of metabolic pathways was also seen in the most severely hypoxic patients (139). Overall, early humoral activation is prominently tied with hypoxia.

Boothby and colleagues found that the GC is hypoxic as GC B cells robustly stabilize Hif-1α and positively stain PIMO based hypoxia dyes (128). They also found that overexpression of Hif-1α in B cells led to impaired antigen specific B cell responses. This was achieved through an adoptive transfer study utilizing B cells lacking pVHL following induction with tamoxifen (and had subsequent overexpression of Hifs). The authors noted a decrease in antibody titers, decreased NP-binding GC B cells, and impaired affinity maturation after immunization with NP-OVA. This was attributed to B cell intrinsic overexpression of Hifs, as pVHL mice crossed to Hif1α^fl/fl^ and Hif2^fl/fl^ mice restored normal B cell function. While this may not reveal the exact physiological role of Hifs in B cells, as the artificial stabilization of Hifs leads to expression in both physioxic and hypoxic conditions as well as potential accumulation of supraphysiological Hif levels, it is clear Hif levels need to be properly maintained within B cells for normal function (128).

Of note, there is some disagreement between as how hypoxia influences CSR of B cells. The Boothby group showed that hypoxia reduces both B cell proliferation and CSR, while the Sitkovsky group suggests that hypoxia promotes these factors. One potential discrepancy between these two claims is the timeframe in which each class switch assay was conducted in hypoxic incubators. Both Cho et al. (128) and Abbott et al. (127) agree that at the late CSR cell culture time point of day 4 hypoxia suppresses B division and CSR rates for B cells. However, in Abbott et al., we additionally evaluated CSR rate and B cell proliferation on days 1, 2, and 3 of hypoxic culture. This examination of early time points revealed that CSR kinetics are actually accelerated and terminal plasma cell formation is enhanced in hypoxic conditions. This suggests that the timing of the B cell response relative to hypoxia may yield a differential response. Relating to GCs, this may be of some significance. It would be interesting to posit that as GCs are forming, they are developing a hypoxic microenvironment which may in fact promote early events observed in GCs such as CSR and development of a pro-apoptotic environment. Moreover, the interesting finding that hypoxic cultures accelerated proliferation of B cells would be in line with the general feature of *in vivo* GCs being both hypoxic and containing highly proliferative B cells that are among the fastest dividing cells known dividing approximately every six hours (137).

Another interesting finding from the Boothby study is that Hif-1α appears to negatively regulate mTORC1 activation in activated B cells (128). This study also suggests that hypoxia within GCs is limited to the LZ where they observe limited PIMO staining by histological analysis.

In contrast, we showed PIMO staining throughout the GC histologically (127) (and unpublished data R.K.A). This is consistent with earlier histological analysis of human tonsil GCs in which Hif-1α appears to be ubiquitously expressed throughout the GC (140). The disparity between oxygen tensions that PIMO can detect (~1.2% O_2_) vs the <3% required for Hif stabilization (2) suggests that PIMO staining may underestimate the true level of hypoxia within the GC microenvironment. Of note, both splenic GCs that were induced following immunization with NP-OVA, as well as constitutively present GCs in Peyer’s Patches and mesenteric lymph nodes, all showed prominent PIMO staining. This suggests that regardless of antigen or location (e.g. spleen or lymph node), hypoxia appears to a be a general feature of GCs. We further validated GC hypoxia by histological measurements showing that much of the area of the GC is beyond a 40-micron distance from perfused vasculature, a known distance in which hypoxia can develop (141). Nevertheless, GC B cells likely experience a range of low oxygen tensions. A recent paper demonstrated that B cells *in vitro* respond differently to oxygen tensions at 1% and 3% (138). While this may not be completely reflective of what occurs in the GC, it warrants further investigation. With oxygen tensions in SLOs ranging from 4-46 mmHg (142) there is also evidence to suggest that these lymphocytes are constantly in a low oxygen environment.

We also demonstrated that hypoxia is an important parameter for GC physiology (127) *in vivo*. Reversing hypoxia by exposing mice immunized with NP-OVA or NP-CGG/Alum to hyperoxic air (60% O_2_) during the first eight days post immunization inhibited the GC response. Hyperoxia significantly reduced GC B cell frequencies along with impairing Tfh formation and their costimulatory potential when compared to mice in normoxic incubation, suggesting hypoxia is an important parameter for the development of a GC reaction. Exposure to hyperoxia also significantly reduced ASC formation, CSR frequency, and resulted in lower antigen specific serum antibody titers. Altogether we found that an oxygen-deprived microenvironment is required for proper GC formation.

Around the same time as the Boothby and Sitkovsky studies, Rickert’s group also independently demonstrated that the GC was hypoxic (129). In Jellusova et al. they showed prominent histological staining of PIMO within GCs and western blotting from purified GC B cells showing robustly stabilized Hif-1α (129). It was also demonstrated that GC B cells face increased metabolic demands as they present with increased protein and mitochondrial content as well as an increased uptake of glucose (129). GC B cells were found to be sensitive to inhibition of glycolysis, as treatment with 2-DG significantly reduced the frequency of GC B cells in immunized mice. These metabolic compensation measures support GC B cell proliferation in response to hypoxia. Furthermore, in Jellusova et al. they also found that the glycogen synthase kinase 3 (GSK3) protein was integral for the GC reaction. Through a series of elegant genetic experiments, GSK3 was found to limit cell mass accumulation and B cell proliferation. When GSK3 is lost in an inducible knock-out system (hCD20-Tam^Cre^ Gsk3a^fl/fl^Gsk3b^fl/fl^), GC B cells were almost completely ablated, and affinity maturation was severely dampened (129). GSK3 was shown to limit B cell proliferation and metabolic activity in response specifically to CD40 stimulation, suggesting that this factor is a more specific regulatory factor for GC B cells (129). Interestingly, GSK3 was shown to interact with other prominent GC transcription factors c-Myc and mTORC1 (129). GSK3 knock-out decreases the sensitivity of B cells to rapamycin and prevents c-Myc degradation, reinforcing a role of GSK3 in restricting proliferative potential in activated B cells (129). Inversely, GSK3 enhances B cells survival when glucose is sparse as seen by decreased viability in GSK3-deficient B cells in culture conditions without glucose (129). Concomitant with decreased viability, GSK3-deficient B cells also accumulated more ROS than WT controls *in vitro*. While not directly tested, it could therefore be hypothesized that GSK3 plays a regulatory role in hypoxic microenvironments by promoting cellular viability in harsh conditions while also preventing excessive proliferation.

GC B cell metabolism becomes increasingly interesting when put in the scope of hypoxia. Metabolic reprogramming was found to be important for GC B cells as expression of Bcl6, the master transcription factor for GCs, was found to be tied to IL-4 signaling and accrual of α-ketoglutarate (αKG) (143). αKG behaves as a cofactor for the UTX demethylase which removes the repressive H3K27me3 epigenetic modification from the Bcl6 locus, potentiating Bcl6 expression (143). Cellular capacity for glucose uptake has historically been assessed by uptake of the fluorescent glucose analog 2-[N-(7-nitrobenz-2-oxa-1,3-diazol-4-yl)amino]-2-deoxy-d-glucose (2-NBDG). GC B cells appear to readily uptake more 2-NBDG compared to non-GC B cells after this compound is injected in immunized mice (129, 144). When B cells are positively selected by Tfh, they begin to undergo a proliferative burst (145). This burst is dependent on glycolysis to meet the energetic requirements of synthesis and division as suggested by their transcriptional upregulation of glycolytic potential and low basal OCR (146). This glycolytic paradigm and overall GC B cell function is supported by three major transcription factors: Hif-1α, c-Myc, and mTORC1. c-Myc and mTORC1 signaling is most prominent in the GC in a subset of LZ GC B cells that have recently received Tfh mediated positive selection signals (145). As the B cells migrate to the LZ to compete for antigen, they may switch from a glycolytic focus to one focused on OxPhos as suggested by their near maximal OCR (146). Recent studies have highlighted a potential role for OxPhos in the GC reaction (147, 148). Transcriptomic analysis of GC B cells reveals an increase in OxPhos transcripts within affinity matured GC B cells (147). This suggests that more competitive GC B cells upregulate OxPhos genes. Another study found that transcription factor A, mitochondrial (TFAM) which is a vial regulator of mitochondrial homeostasis and function, is required for proper GC development (148). When TFAM factor is lost in specifically in GC B cells using an aicda-Cre (gene encoding AID) Tfam^fl/fl^ system there is a severe restriction in the GC B cell and antigen specific GC B cell compartments (148). This finding coupled with the inhibition of GC B cells by 2-DG treatment suggests that GC B cells utilize multiple metabolic pathways to meet their energetic needs within their hypoxic microenvironment. A study by Iborra-Pernichi et al. intensively studied GC B cell metabolism with B cell sufficient and deficient in TFAM (Mb1-cre TFAM^fl/fl^) (149). In alignment with previous work, mice with B cells deficient in TFAM presented with lower GC B cell frequencies after immunization. TFAM deficient B cells did display a lower OCR (upon stimulation) and mitochondrial dependency (in GC B cells) than the WT controls but were able to compensate for ATP production by upregulation of various other metabolic pathways. GC frequency defects in response to TFAM loss were attributed not to failure of meeting bioenergetic requirements, but rather lack of proper mitochondrial remodeling that impaired lysosomal degradation of antigen. The authors suggest that this dysregulated mitochondrial-lysosomal axis prevents adequate antigen presentation of GC B cells to their cognate Tfh which impairs the GC reaction. While a full appreciation of metabolic profiles of GC B cells in each zone remains elusive, there has been significant progress in elucidating GC B cell metabolism.

GSK3 inhibition coincided with an accumulation of the pro-proliferative transcription factor c-Myc as well as signaling molecules downstream of BCR engagement (129), suggesting GSK3 is inhibited in LZ GC B cells undergoing positive selection. The Victora group completed a set of very elegant studies to understand how metabolic signals through mTORC1 affect selection events in GCs (145). As LZ B cells present antigen to a cognate Tfh, they receive survival signals such as CD40L and IL-21. Following this positive selection, LZ GC B cells upregulate mTORC1 (145) that is concomitant with c-Myc expression. Positively selected LZ B cells then then return to the DZ. This increase in mTORC1 gives the successful clone the ability to generate enough biomass to undergo multiple rounds of division (145). Each successive division within the DZ lowers the amount of mTORC1 present in the daughter cells, until there is a low enough level of mTORC1 present to move into the LZ once again. Overexpression of mTORC1 confined B cells to the DZ of active GCs, demonstrating the unique role mTORC1 plays on the DZ phenotype and physiology (145).

A recent study claims that GC B cells minimally utilize glycolysis, while predominantly using fatty acid oxidation for energetic demand (150). However, this finding is unsupported by the body of evidence concerning GC metabolism, particularly how inhibition of glycolysis with 2-deoxy-d-glucose (2-DG) inhibits GC reactions in multiple model systems (129, 151, 152) as well as severe reduction of GC B cells when GSK3α/β is selectively or temporally deleted in B cells using various Cre systems (129). Additionally, a recent study of B cell intrinsic deletion of the GLUT-1 transporter showed reduction in the frequency of GC B cells (153) following immunization and at steady state suggesting glycolysis as a vital pathway for the GC. Moreover, the claim that GCs do not utilize glycolysis principally relies on *ex-vivo* restimulation of GC B cells in a seahorse assay following purification, all while the cells are held under supraphysiological oxygen conditions (21% O_2_) (150) (**Figure 1**). Of note, Hif-1α has been detected by multiple groups to be stabilized in GC B cells (128, 129, 146), and is notoriously unstable under normoxic conditions (29). In fact, Hif-1α has a half-life of less than 5 minutes in normoxic air (7), leading to infamous detection challenges utilizing standard methods. This raises considerable doubt about whether such sweeping conclusions can be drawn principally from *ex vivo* experiments utilizing GC B cells in non-physiological cell culture conditions and whether this is reflective of the true metabolic state of GC B cells *in vivo*. Boothby and colleagues made several salient points concerning the technical limitations of this study (154).

There have been various investigations into how GC cells react and adapt to hypoxic environments. Mice exhibiting constitutive stabilization of Hifs through pVHL deletion had lower levels of antibody in the serum as well as lower affinity antibodies, lower antigen specific GC B cells (128), and lower expression of key GC transcripts. This demonstrates that precise control of Hif-1α expression is vital for a successful GC reaction. Despite this profound impact on the GC response, Hif-1α is not required for the activation of B cells *in vitro* while the transcription factor c-Myc is (155). Genetic deletion of Hif-1α in all hematopoietic cells using a tamoxifen inducible Rosa26-CreERT2-Hif-1α^fl/fl^ system results in multiple defects in the GC compartment post immunization with NP-OVA. These include pronounced reductions of antigen specific GC B cells, Tfh numbers and function, altered Tfh/Tfreg ratio, and serum antibody levels (144). Utilizing the same system, when both Hif-1α and Hif-2α were deleted, a similar response was noted (144). Transfer of CD4 T cells from either Rosa26-CreERT2-Hif-1α^fl/fl^ or Rosa26-CreERT2-Hif-1α^fl/fl^-Hif-2α^fl/fl^ into TCRα KO mice revealed that these defects are at least in part T-cell intrinsic.

Adaptation to hypoxia is not solely restricted to changes in cellular metabolic programs. The hypoxia responses also elicit a shift in global protein expression. When cultured in hypoxic conditions, human B cells upregulate expression of the chemotactic receptor CXCR4 (156). CXCR4 expression also correlated with increased B cell viability in hypoxic culture, and its expression was found to be controlled by both Hif-1α and Nrf2 (156). Nrf2 is a transcription factor that upregulates protective factors against reactive oxygen species, serving a similar function to Hif-1α in upregulating survival mechanisms in specific conditions. CXCR4 is of particular importance in the humoral immune response. In conjunction with its ligand CXCL12, CXCR4 helps to differentiate the DZ of the GC (157) as well as the specialized bone marrow niche in which LLPCs reside (158). Nrf2 has also been shown to interact with Hif-1α and Syk for control ROS production and B cell viability in hypoxic conditions (159). The mitochondrial Na/Ca exchanger NCLX is also important for maintaining cellular function in hypoxia. When NCLX is lost in B cells by a CD23-Cre Slc8b1^fl/fl^system there is a decrease in the mobility of Ca^2+^ in activated B cells which measurably decreased the number of cells in the GC reaction (160). While there were defects seen in GC frequency, the amount of antigen-specific antibody displays no difference but there is a significant difference in the amount of total antibody present in the serum (160).

Hif-1α also mediates upregulation of TASK-2 K+ channels that increase Ca^2+^ signaling in B cells during hypoxic stress (161). Sustained hypoxia induced hyperpolarization of the cell membrane which could only be reversed by high K+ depolarization or silencing of the TASK-2 mRNA (161). This is thought to be important in maintaining negative potential to provide sufficient electrochemical driving force to control calcium influx upon BCR stimulation (161). Both Task2 and mitochondrial NCLX maintain membrane potential and ionic balance in stress conditions. Mitochondrial biomass was increased upon activation with either BCR crosslinking (by anti-IgM antibody) or TLR9 ligation (by unmethylated CpG oligonucleotides). Without the canonical second signal, toxic ROS and Ca^2+^ accumulates in the activated B cell causing extreme mitochondrial dysfunction leading to cell death (162). This was directly linked to the strength and duration of BCR engagement (162).

Proper Tfh differentiation is reliant on Hif-1α (163). When Hif-1α is lost, there is a significant reduction of this population (163). This differentiation is also reliant on glycolysis as when this pathway is blocked with 2-DG, there is a dramatic decrease in this compartment (163). Both Tfh and GC B cells appear to require glycolysis. This is confirmed when a key glycolytic enzyme, lactate dehydrogenase (LDHA), is lost there is substantial reduction in both GC B cells and Tfh cells (164). When LDHA is conditionally deleted in a CD4-cre system, Tfh without LDHA display lower help function, resulting in diminished GCs (164). When B cells lose LDHA by a conditional deletion by CD23-cre, they do not activate correctly and display a severely reduced GC formation (164). This reliance may be temporal, as when LDHA is lost upon transcription of the Aicda locus there is a lesser defect in total GC number and affinity maturation (164). The discrepancy between CD23-cre and AID-cre systems may be in part due to limitations of the AID-cre system. AID has long been known to modulate B cell susceptibility to death (165). One limitation of the AID-cre system is that it is knock-in/knock-out, removing one copy of the AID gene (166). This may allow for a potential compensation for the deleterious loss of LDHA with a rescue phenotype from the hemizygous loss of AID, as AID is known to promote apoptosis of GC B cells (165) independent of its roles in CSR and SHM.

Of note, when human plasma cells are cultured in hypoxic conditions, there was an increase in plasma cell gene signatures and antibody production (136). Interestingly, another study found that secreted metabolites from mesenchymal stromal cells dramatically increase the viability of plasma cells and increase the titer of antibodies they can produce. Using the secretome of these mesenchymal stromal cells, APRIL, and culturing these cells in hypoxic conditions had the most pronounced increase in effector function and cell longevity (167), demonstrating again that normoxia may be the suboptimal condition of this cell type. This also demonstrates that humoral responses are heavily reliant on both physiological conditions surrounding their niches but also signals/metabolites in the local milieu to perform their functions.

In sum, multiple studies have revealed that the GC develops a hypoxic microenvironment that is important for its function (127–129, 144). In fact, hypoxia appears to be intrinsically tied to the humoral response at almost every level of development (81, 85, 86), activation in GC reactions (127–129, 144), and terminal differentiation/effector function of plasma cells (127, 136, 167). While great progress has been made in understanding the effect of hypoxia on B cells in relation to their physiological role, there is still much to elucidate to fully appreciate the impact hypoxia has on normal and pathological B cell responses.

## Role of the hypoxia-adenosinergic pathway in regulating humoral immune responses

**Figure 4:**
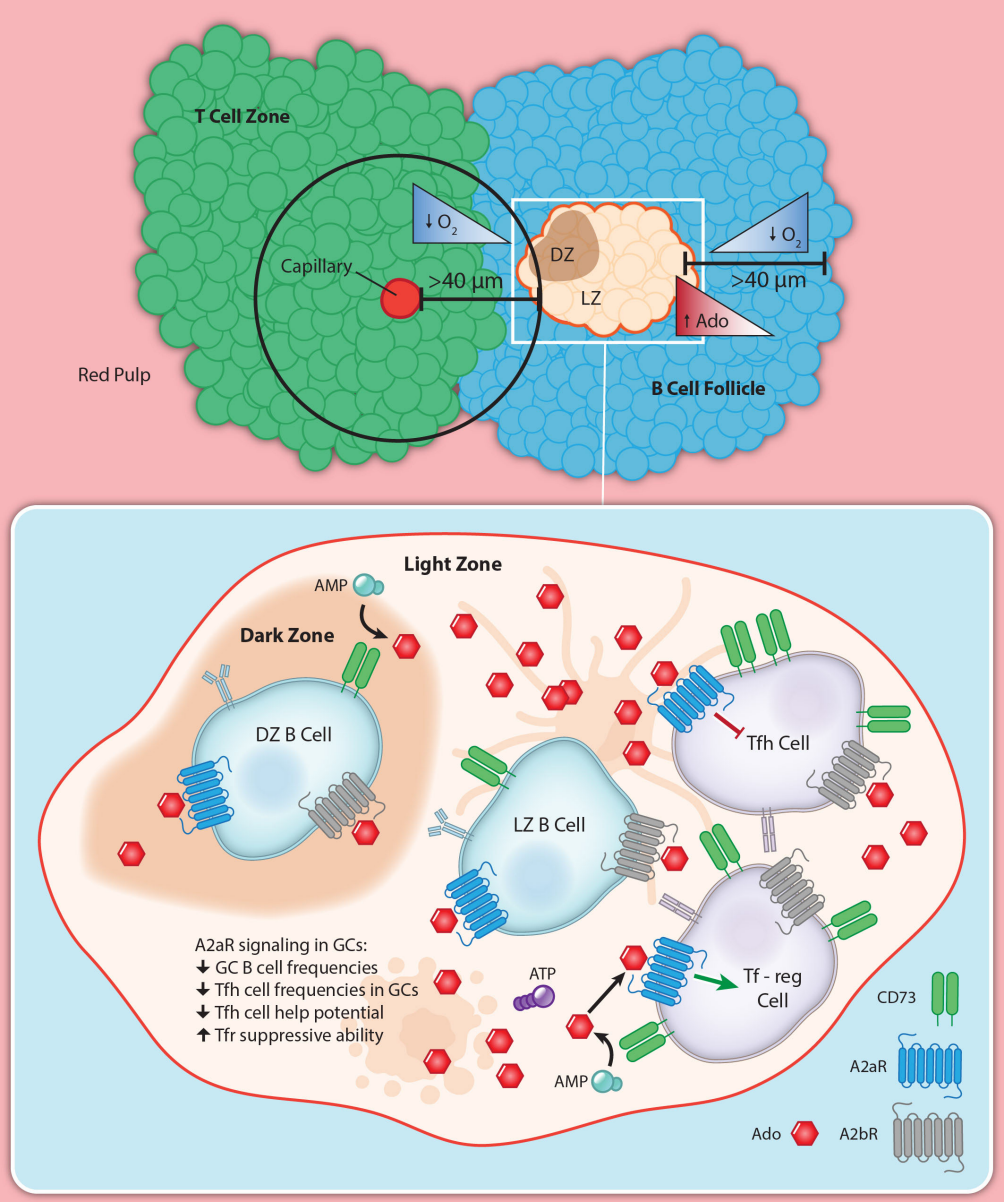
Hypoxia-adenosinergic regulation of germinal centers. GCs often form in the middle of B cell follicles in SLOs. This localization puts a majority of the GC area >40 μm away from the nearest perfused tissue, which potentiates a hypoxic microenvironment. The hypoxic microenvironment develops pro-adenosinergic phenotype such as CD73 upregulation on GC B cells and high expression on Tfh. A2aR has been shown to limit GC reactions globally, and is at least in part due to direct A2aR regulation of Tfh help.

The GC is the heart of the humoral response. The microenvironment of the GC makes it a prime candidate to be under the influence of the hypoxia-adenosinergic pathway (**Figure 4**). GC B cells, once activated, undergo a cell division once about every six hours (137, 168), making GC B cells among the fastest dividing mitotic cells in the body. This high rate of cell division is coupled with an extremely competitive selection process, as the majority of GC B cells die by apoptosis (113). While direct measurements of the extracellular space within GCs is not readily feasible, it is plausible that the high rate of apoptosis yields a high amount of extracellular ATP in the GC microenvironment. Facilitating degradation of ATP into adenosine, GC B cells have been shown to increase in CD73 expression over time (15, 169), while globally B2 cells constitutively express CD39 (112, 170). CD19^+^ extracellular vesicles have been characterized to also have these ectoenzymes on their membranes (171) suggesting small apoptotic bodies can also catabolize ATP down to adenosine. Tfh cells have also been found to express very high levels of CD73 (15), while Tfregs express CD39 which may further contribute to the creation of eAdo. It is reasonable to posit that the GC microenvironment is rich in extracellular adenosine.

The hypoxia-adenosine axis has been shown to regulate GC responses (15, 17, 20). The Sitkovsky group found that in addition to hypoxia, GCs appear to develop a pro-adenosinergic microenvironment. GC resident follicular T cells were shown to express high levels of CD73. Additionally, purified follicular T cells showed high levels of A2aR signaling capacity bya substantial accumulation of cAMP when stimulated with the A2aR-specific agonist CGS21680, compared to control CD4 T cells. Immunization of mice with germline deletion of A2aR resulted in exaggerated GC responses, suggesting A2aR signaling serves to restrain GC reactions *in vivo*. T cell occupancy within GCs were also greater in these knock-out mice (15, 20). CSR was also remarkably more frequent in mice that lacked the A2aR. These mice also demonstrated increased Tfh frequencies, number of T cells within GCs with a greater costimulatory potential presenting with higher ICOS expression. Tfregs also displayed reduced immunosuppressive CTLA-4 expression in A2aR-KO mice. A greater number of T follicular cells also expressed the proliferation marker Ki-67 in response to loss of A2aR. Through an adoptive transfer system, the investigators demonstrated that A2aR impacts the GC, at least in part in a T cell intrinsic influence manner, on the GC. When T cell deficient mice are given A2aR-KO T cells there is a greater GC response and Tfh cell outgrowth over WT controls after NP-OVA immunization. This confirmed the cell-intrinsic contribution of T cells to the GC by the hypoxia-adenosinergic pathway.

The Mueller group also identified T cell intrinsic hypoxia-adenosinergic contribution to the GC (20). They found that treatment with the A2aR-specific agonist CGS21680 repressed Tfh formation in response to 2W1S-PE immunization in mice. They also demonstrated that this repression is directly tied to an increase of expression of the RORγt transcription factor, driving T cells toward a Th17 phenotype. Additionally, Tfh phenotype was reduced in A2aR agonized mice. These effects were a consequence of A2aR signaling, as these shifts were not seen in A2aR-KO T cells treated with an A2aR agonist. In agreement, CGS21680 treated WT mice displayed impaired GC and antigen-specific responses. This impairment was not seen in A2aR-KO mice post treatment. Subsequent study utilizing an innovative transfer based autoimmune model of arthritis demonstrated that A2aR agonism significantly impairs the formation of auto-immune Tfh in GCs and prevents establishment of arthritis in CGS21680 treated mice (19). In line with this, A2aR has been shown to be a prominent target in regulating antibody mediated autoimmune disorders. A subset of CD11c^+^Tbet^+^ B cells have been shown to be contributory to systemic lupus erythematosus (SLE) (13). These CD11c^+^Tbet^+^ B cells also demonstrate higher expression of the A2aR compared to CD11c^-^ B cells (172). Treatment of mice experiencing SLE with A2aR agonist resulted in a significant reduction in these pathogenic CD11c^+^Tbet^+^ B cells and reduced pathology (13). Interestingly, treatment with an A2aR agonist also significantly reduced the Tfh and plasma cells in the spleen and lymph nodes of these autoimmune mice (13). This data further suggests that the hypoxia-adenosinergic pathway plays a role in regulating humoral immunity. Specifically, A2aR signaling is immunosuppressive in the humoral response and can act as a viable target for regulating autoimmune disorders. These findings indicate A2aR signaling serves to restrain GC reactions, which can be detrimental for vaccine responses or beneficial for treating autoimmunity.

Further evidence of eAdo acting as a suppressive molecule in GC reactions comes from studies exploring the impact of adenosine deaminase (ADA) as a regulator of immunity. ADA reduces adenosine to inosine which largely ablates adenosinergic signaling. This protein was found to be required for Tfh differentiation (16). When ADA is absent there are dramatic defects in Tfh differentiation and aberrant GC architecture leading to immunodeficiencies, suggesting there is a pathological effect to letting Ado accumulate (16). Curiously, when given as a component of a vaccine, ADA amplified the magnitude of Tfh engagement and augmented the downstream products of the GC reaction including an increase in antigen specific antibody and an increase in durability of the vaccine response (17, 18). Recently, the low-affinity Ado receptor A2bR has been implicated as a unique functional marker for a subset of highly efficient human Tfh cells (16). It was also found by the Haddad group that HIV impacts GC Tfh by the downregulation of c-Maf (21). Excitingly however, through exogenous supplementation of ADA within GC-Tfh and GC B cell co-cultures, they demonstrated rescued c-Maf signaling capacity. This was evidenced by upregulation of the IL-6 pathway downstream of c-Maf within chronically HIV infected lymph node cells (21). This offers valuable insight in potential translational mechanisms surrounding adenosinergic signaling. Overall, it appears that adenosinergic signaling is vital for GC populations.

Another link between eAdo and GC populations is the relationship between expression of ecto-enzymes CD39/CD73 and proper development and activation of lymphocytic populations. As mentioned previously, CD73 increases in expression in the GC as it matures (15, 169) but this expression was found to be a vital condition for the correct differentiation and maintenance of ASCs. There is a significant diminishment of ASCs produced in the late primary response post NP immunization when CD73 is deleted (169). There is a severe reduction in ASCs in bone marrow chimeric mice that receive CD73 deficient lymphocytes (169). This reduction is not seen when CD73 is lost exclusively on either B or T cells independently, but is only seen when CD73 is deleted on both B and T cells. CD39 and CD73 also appear to control Tfh generation. When these ectoenzymes are neutralized *in vitro* under Tfh differentiating conditions there is a more robust generation of this cell population (173). These findings are particularly interesting since both B and T cells appear to have the ability to produce eAdo but have different cell intrinsic responses to the eAdo produced. This may suggest the importance of a dynamic eAdo concentration at various points in time to orchestrate a proper, balanced immune response.

In sum, multiple studies have revealed that the GC develops a hypoxia-adenosinergic microenvironment as eAdo signaling has also been shown to be critical for regulation of GC responses, principally through the A2aR. At least part of A2aR mediated GC regulation is T cell intrinsic (15–18, 20). Little exploration has been done to determine whether a cell intrinsic impact exists on GC B cells despite their prominent role in extracellular nucleotide catabolism (77, 169, 173). Future studies could utilize these conserved hypoxic pathways to pharmacologically enhance humoral immunity and help generate robust protection from evasive pathogens such as influenza or HIV.

## Role for hypoxia and metabolic signaling in T cell development

Thymic tissue appears pale and poorly vascularized (174) making it an obvious place to evaluate whether tissue hypoxia played a role in the biology of T cell development (**Figure 3**). The first detailed report of hypoxic regions being present in the thymus came from Dewherst and colleagues using PIMO probe as well as direct measurement of oxygen tension by platinum micro-electrode probing (174). Areas in the thymus containing strong PIMO staining clearly clustered in areas 50 microns or more from defined blood vessels, although these distances were not directly assessed. However, in confirmation that this PIMO staining is directly dependent on oxygen tension, some mice were subjected to hyperoxia (breathing 100% O_2_ for 4 hours before tissue harvest) as a control. As expected, PIMO staining in these mice was substantially reduced after hyperoxic respiration. Quite curiously, several hypoxic responsive genes (BNIP3, HMOX-1) were not induced in thymocytes compared to control tissues of lung and spleen (174). While this may be a technical limitation due to issues such as purification of cells, the authors posit that this result may be due to the physiology of the tissue. Specifically, if the thymus is a hypoxic microenvironment at steady state, then under a state of chronic hypoxia certain response genes may not be induced. In the case of the thymus, this may result in preserving a pro-apoptotic environment necessary for proper thymocyte development.

In line with this rationale, a genetic study found that when the gene encoding the pVHL is conditionally deleted in thymocytes, there is a significant loss of double positive thymocytes (175). The study found no difference in the number or distribution of double negative thymocytes, alluding that the impact of losing Hif-1α regulation in this model was at the double positive selection events (175). Accordingly, there was a significant upregulation of caspase 3 activity and terminal deoxynucleotidyl transferase dUTP nick end labeling (TUNEL) in thymocytes with rampant Hif-1α activation, potentiating the loss of cellularity to Hif-1α induced apoptosis (175). This was attributed to Hif-1α dependent decrease in expression of the BCL-XL survival signal known to be vital for double-positive thymocytes (175, 176). All these factors support the notion that thymic hypoxia promotes a strict environment that ensures proper T cell development.

## Role for hypoxia and metabolic signaling in cellular mediated immunity

In T cells, Hif-1α stabilization promotes anaerobic glycolysis by upregulating key glycolytic enzymes and glucose transporters (177). Effector T cells tend to use glycolysis for their energetic needs, while memory/regulatory T cells tend to utilize OxPhos (177). Hif-1α stabilization promotes the expansion of effector populations such as the proinflammatory Th17 subset. Conversely, loss of Hif-1α reduced Th17 numbers while enhancing Treg populations (178). This shift in cell populations was found to be dependent on both mTORC and glycolysis. Pharmacologic inhibition of glycolysis or mTORC signaling *in vitro* impaired the development of Th17 cells. Rapamycin inhibition of mTORC also demonstrated a pronounced decrease in expression of Hif-1α (179). Hypoxic expansion of Th17 cells is thought to be attributed to direct binding of Hif-1α to the RORγt locus. ChIP-seq analysis revealed that Hif-1α binds the promotor region of RORγt (180). Hif-1α and RORγt were also revealed to create a complex to amplify the expression of Th17 genes such as IL-17F and IL-23R (180). Th17 cells also display a higher persistence *in vivo* when compared to other Th subsets. This is potentially owed to Hif-1α inducing the expression of the Notch signaling pathway which upregulates the Bcl-2 family of proteins, a known repressor of apoptosis (181). CD4^+^ T cells are sensitive to inhibition of glutamine metabolism. Pharmacological inhibition of glutamine metabolism results in a severe decrease in the ability of these cells to produce cytokines and lowers the expression of critical receptors like CCR6 and CXCR3 (182). Endogenous glutamine levels have been shown to be a rate-limiting substrate for activation of CD4 T-cells under hypoxic conditions (183). When glutamine metabolism is pharmacologically inhibited there is a dramatic decrease in the OCR and ECAR, activity of enzymes involved in these pathways, and proliferative potential (183). Overall, Hif-1α governs many vital cellular processes that influence function of effector T cells, as observed notably in Th17 cells which are often found at sites of inflammation (184).

Another mechanism for hypoxic control of T cell differentiation is the upregulation of FoxP3 (185, 186). Hif-1α promotes expression of FoxP3 and facilitates a more robust Treg population (185, 186). Another study found that Hif-1α induced and controlled early metabolic functions required for Treg1 differentiation. This was attributed to upregulation of the ectoenzyme CD39. CD39 then depleted extracellular ATP, which promoted the Treg identity *in vivo (* 187). Hypoxic induction of Tregs is thought to prevent excess inflammation (185, 186). When Hif-1α is deleted in T cells by a CD4-Cre Hif-1α^fl/fl^ system, resultant mice show an appreciable increase in systemic inflammation, as well as display an upregulated Th17/Th1 and Th17/Treg ratio (188, 189). This demonstrates that Hif-1α is involved in the restriction of aberrant T cell inflammation.

Additionally, an exacerbated inflammatory response with elevated IFN-y production is seen in mice with constitutive Hif stabilization in Tregs (190). This is in line with previous work demonstrating that CD4^+^ T cells activated *in vitro* and cultured at hypoxic conditions display a significant increase in the amount of IFN-γ in the supernatent (46). Tregs that have lost the ability to control Hif-1α by a FoxP3-cre Vhl^fl/fl^ system also have reduced regulatory function, allowing more pronounced Th1 effector function in these mice (190). Interestingly, another interaction with Hif-1α and FoxP3 has been identified. Some studies suggest that Hif-1α can bind directly to FoxP3 and facilitates degradation and loss of Treg identity (180, 191). This negative regulation between Hif-1α and FoxP3 has been reviewed by the Lai group (192). While these two methods of Hif regulating Tregs appear juxtaposed, there may be unidentified factors the contribute to hypoxic regulation of Tregs that are responsible for this discrepancy in the field. Interestingly however, mice bearing lung tumors housed in hyperoxic conditions (60% O_2_), weakening the hypoxia-adenosinergic signaling axis, demonstrated a reduced number of tumor infiltrating Tregs and a lower expression of FoxP3, CD39, CD73 and CTLA4 compared to mice in normoxic conditions (193). These data demonstrate that hypoxia and Hif-1α play a vital role in T cell activation and differentiation, acting as a regulator of cellular responses.

Hif-1α was found to be essential for CD8^+^ T cell function. There is a loss of glycolytic expression that is required for proper differentiation when Hif-1α is deleted in T cells (194). Concomitant with this, there is a downregulation of the production of cytotoxic enzymes, and a decrease in display of receptors associated with T cell-mediated tumor rejection (194). Hif-1α stabilization in CD8^+^ T cells by pVHL deletion results in an enhanced glycolytic potential. These T cells showed an increase ability to control infection and cancer and have greater exhaustion refractory potental (195). Hypoxia also exacerbates hyperresponsiveness and inflammation in CD8^+^ T cells in a Hif-1α dependent manner (196). A detailed study of comparative gene profiles in T cells responding to gliomas demonstrated that T cells with a higher level of Hif-1α expression were found to be closely linked to a more exhausted profile, while T cells that had a lower Hif-1α expression maintained an effector profile (197). This could be suggestive of a dose-dependent response to Hif-1α in modulating the difference between effector cells and exhausted cells. Another potential interpretation is that the tumor microenvironment creates a complicated extracellular milieu that could not be replicated in studies above.

Hypoxia also appears to play a role in recall responses. Curiously, restimulation of CD8^+^ memory T cells results in upregulation of rapamycin-insensitive glycolysis (198). This suggests that the primary metabolic response was “imprinted” in the memory cells. Mechanistically, this was proposed to be due to the uncoupling Akt from mTORC to increase glycolytic potential (198). Memory CD8^+^ T cells may encounter hypoxia and begin to engage Hif-1α dependent glycolytic programs to induce a shift toward an effector phenotype. Memory CD8^+^ T cells cultured in hypoxic conditions displayed an enhanced effector phenotype and glycolytic potential over cells cultured in hyperoxia (199). Investigators suggest that this may be a viable method to reinvigorate memory CD8+ cells prior to immunotherapies. A study by the Yee group found that hypoxia and TGF-β1 are vital milieu queues that induce a tissue resident memory (T_RM_) phenotype in CD8^+^ T cells (200). Isolated human CD8^+^ T cells cultured in hypoxic conditions more robustly adopted a canonical T_RM_ CD69^+^CD103^+^ phenotype and acquired a more authentic T_RM_ transcriptomic profile than those cultured in normoxic conditions (200). Overall, these findings establish hypoxia as an influencing parameter in cell mediated recall responses.

Hif-1α does not solely impact T cells by altering dominant metabolic pathways. Hif-1α induces an epigenetic reprogramming of CD8^+^ T cells that leads to an accumulation of S-2-Hydroxyglutarate (S-2HG). This reprogramming was found to be highly influential for CD8^+^ T cell fate, as exogenous treatment with S-2HG dramatically increased proliferation potential, anti-tumor function, and lowered exhaustion (201). The PDK1-Akt-mTORC1 axis was also found to be indispensable for the efficient differentiation into effector CD8^+^ T cells. This pathway is downstream of Hif-1α activation, and regulates trafficking, expression of glycolytic enzymes, expression of cytotoxic receptors and effector molecules (202). Glycolysis leads to acute and sustained effector activation in T cells. When glycolysis is abrogated by 2-DG treatment in activated CD8^+^ T cells, they will begin to utilize fatty acid oxidation for their energy demands. This metabolic shift pushed their phenotype into a more memory-like state. When T cells are expanded *ex vivo* in the presence of 2-DG they display greater antitumor function upon infusion. When these T cells expanded without utilizing glycolysis are adoptively transferred into a tumor bearing mouse there was a greater reduction in tumor size and increased inflammatory cytokine production (177). In line with metabolism dictating function, there are dramatic differences noted when either mTORC1 or mTORC2 is differentially regulated. When CD8^+^ T cells lose the ability to regulate the mTORC1 complex by Tsc2 deletion, there is an increase in terminally differentiated T cell populations. Rapamycin treatment recovers wild-type distributions of T cell populations, denoting that this is causally tied to the mTORC complex (203). mTORC2 is seen to negatively govern the establishment of the memory phenotype in T cells. When Rictor is deleted within T cells there is no loss in effector function but there is an increase in the emergence of memory T cells (204). mTORC2 seems to play an interesting role as when this complex is lost but mTORC1 is hyperactive, there is an enhanced rate of cell death (203). This suggests that manipulating metabolic programming could have an impact on cellular therapeutics. Better understanding of the dynamic metabolism-to-phenotype connection could dramatically alter how cell-based therapies are designed.

When Hif-1α deletion is restricted to the T cell compartment utilizing CD4-cre Hif-1α^fl/fl^ system, a severe restriction of GC outgrowth is noticed following immunization (144). This suggests that Hif-1α in T cells is at least partially contributory to the success of the GC reaction (144). Interestingly, loss of Hif-1α alone in T cells did not impact the development of high affinity antibodies against the immunogen, but loss of both Hif-1α and Hif-2α in T cells impaired the development of high-affinity class-switched antibodies (144). It is worth noting that this was not analyzed in mice with T cells deficient solely in Hif-2α (144). This suggests that Hif-2α plays a prominent role in T cells at some point in the humoral response or can compensate for Hif-1α loss (144).

In sum, Hif-1α and its metabolic consequences have dramatic impacts on T cell phenotype and function. Hif-1α expression increases glycolytic potential and pushes cells into an effector phenotype. Conversely, when Hif-1α or glycolysis is blocked, there is an adaptation toward a memory/quiescent phenotype. This reinforces the idea that T cell function is substantially reliant on glycolytic metabolism. Immunometabolism could be a prominent target for prevention of hyperactive or uncontrolled cellular responses.

## Role for hypoxia-adenosinergic signaling in cell mediated immunity

The hypoxia-adenosinergic pathway has been shown to be critical in regulating cell mediated immunity. This pathway has been extensively studied in cancer immunology. This topic has been reviewed elsewhere (205–207), so we will briefly cover the consensus. The hypoxia-adenosinergic pathway characterized by a sequence of physiological changes that occur during inflammation to promote immune regulation. Ectoenzymes are upregulated in various cell types within this hypoxic microenvironment which convert extracellular ATP to eAdo. This eAdo then binds to adenosine receptors on T cells (**Figure 2**). A2aR is the most extensively studied in the context of cell mediated immunity (80, 205, 206, 208, 209) but A2bR has also been demonstrated to modulate T cell responses (210–212). Natural killer cells are also among the highest expressers of A2aR and are dramatically impacted by A2aR targeting therapies (213). Generally, the hypoxia-adenosinergic pathway has been demonstrated to be immunosuppressive in T cells.

Tregs, activated T cells, and Tfh often express both CD39 and CD73 (214). This allows T cells to create and respond to eAdo in the microenvironment. In ischemic hypoxia, the hypoxia-adenosinergic pathway serves to limit tissue damage by repressing the degranulation and limiting secretion of inflammatory cytokines in T cells (215). In infection, hypoxia does not often develop immediately (216) allowing effector cells to respond to the pathogen. As effector functions at sites of local infection take their metabolic toll and hypoxia ensues, the upregulation of CD39 promotes Treg differentiation and limits continued effector function (217). Ideally this coincides with the resolution of infection and prevents excess damage by inflammation.

In relation to cancer responses, the tumor microenvironment is known to contain extremely hypoxic regions. This hypoxic microenvironment has been demonstrated to be highly immunosuppressive for the CD4^+^ and CD8^+^ effector function, leading to exhaustion and anergic phenotypes (14, 218). Additionally, histological analysis demonstrated that CD8^+^ T cells avoid hypoxic tumor areas (193), potentiating a favorable environment for the tumor. Hyperoxygenation therapy results in the downregulation of the immunosuppressive components of the hypoxia-adenosinergic pathways (CD39, CD73, and Cox-2) (63). Innovative blockade of the hypoxia-adenosinergic pathway has demonstrated great potential in reinvigorating the responding T cell population. This has principally been achieved by either antagonism of CD73 and/or A2aR. CD73 has been characterized as an indicator of poor prognosis in solid tumor malignancies (218). Blocking CD73 by mAb treatment increased immunological responses against solid tumors (219). Interestingly, anti-CD73 treatment downregulated CD73 expression on peripheral T cells (219). In a foundational study by Ohta and Sitkovsky, it was demonstrated that mice genetically lacking the A2aR receptor could better control of tumor growth in melanoma and lymphoma tumor models (220). In agreement with these genetic findings, treatment with A2aR antagonists dramatically increased tumor destruction (220). Direct A2aR antagonists have been shown to reverse adenosinergic inhibition of T cells and reducing tumor size (221–224). Combination therapies containing CD39, CD73, and A2aR antagonists also show promise in anti-tumor treatment (218, 225). Overall, the hypoxia-adenosinergic pathway serves to limit immune activation in cell mediated immunity and is an active area of research for cancer therapeutics (inhibition of this pathway) and potentially autoimmune diseases (agonism of this pathway). Investigations in the hypoxia-adenosinergic pathway have resulted in over 15 current clinical trials targeting CD39, CD73, or A2aR as a way to improve immunological responses in various forms of malignancies (218).

## Role of hypoxia and metabolism in hematopoietic stem cell quiescence and differentiation

Hypoxia is involved in lymphocytic development from the earliest stages. B, T, and natural killer cells are derived from HSCs, which reside in the hypoxic niche of the bone marrow. Interestingly, the most pluripotent HSCs are in the most hypoxic (near anoxic) regions of the bone marrow (92), most distal from the vasculature (94). The localization of these cells in this hypoxic zone is thought to be protective, preventing damage or loss of these cells to oxygen radicals. Interestingly, hypoxia enhanced differentiation potential of HSCs. Human embryonic stem cells differentiated in hypoxic conditions *in vitro* yielded greater resultant populations compared to cells differentiated in hyperoxia. Not only were more cells generated in this hypoxic condition, but they also differentiated into subsequent lymphocytic populations at a greater efficiency than the stem cells derived from “normoxic” (20% O_2_) culture conditions (226). Hif-1α was also demonstrated to be required for the quiescence of HSCs, a vital component in keeping these highly valuable cells in a low turnover state (227, 228). When Hif-1α is pharmacologically stabilized, there is a more robust reconstitution of blood populations derived from HSCs after non-lethal irradiation (227, 228). This alludes to the greater pluripotent potential of these hypoxic HSCs to perform their hematopoietic functions. In agreement with this, long term HSCs were found to display low mitochondrial potential compared to other cells within bone marrow (228). HSCs also show a reduced oxygen consumption but have enriched glycolytic flux and expression of Hif-1α (228). Hypoxia appears to be a critical parameter to maintain this cell population.

In an elegant transplant experiment, Hif-1α deficient and Hif-1α competent HSCs were transferred in equal number to lethally irradiated mice. Initially, there is a massive expansion of Hif-1α^-/-^ cells in the peripheral blood and a reduction in LSK^+^ Hif-1α^-/-^ cells in the bone marrow. This demonstrates that when Hif-1α is lost there is a loss of the quiescent state of HSCs. This results in rapid and irreversible differentiation of these pluripotent progenitor cells. This was exemplified by a secondary transfer experiment. HSCs from primary recipients were transferred to another lethally irradiated host. Within this host there was a near complete absence of Hif-1α^-/-^ cells in the blood or BM (229). Absence of Hif-1α^-/-^ cells demonstrates that there were no pluripotent Hif-1α^-/-^ HSCs left in the first recipient, suggesting Hif-1α controls persistence of this cell type. This study also found that Hif-1α was vital to maintain HSC populations in response to physiological stress like aging (229).

It was also recently shown that hypoxia was vital in maintaining the hematopoietic potential of HSCs at various stages of development from HSC to lymphocytes (230). This same study demonstrated that when progenitor cells are cultured in hypoxic conditions prior to transfer, they demonstrate improved functionality post transfer. HSCs cultured in hypoxia show a higher grafting efficiency, improved differentiation potential into all lymphocytic populations (T, B, and NK), and an improved retention of gene signatures of HSCs (230). All these effects were found to be intrinsically tied with either Hif-1α or Hif-2α. When either Hif isoform is silenced by expression of an interfering RNA construct there are dramatic differences in differentiation potentials. Hif-1α was seen to control lymphoid-primed multipotent progenitors (precursors to B/T/NK subsets) while Hif-2α controlled pro-T/NK precursors *in vitro (* 230). Taken together, it is evident that hypoxia, and the metabolic changes it induces, are vital for maintaining pluripotency and functionality of HSCs.

## Hypoxia in the development and function of B regulatory cells

Regulatory B cells (Bregs) are a small fraction of B cells that have been shown to modulate immune responses. Recent studies have seen that Bregs are a subset of B cells that can produce anti-inflammatory cytokines such as TGF-β, IL-10, and IL-35 (231–236). Hif-1α is required to produce IL-10 in B cells (cooperates with STAT3) as when this factor is lost in a Mb1-cre Hif-1α^fl/fl^ model there is a significant reduction in secreted IL-10. However, Hif-1α is not required for other immunosuppressive cytokines like TGF-β or IL-35 (237). IL-10 producing Bregs cells also demonstrate a positive relationship with Tregs. When IL-10 bearing Bregs are reduced so are Tregs, and vice versa (237). OxPhos is required for production of IL-10 and IL-10 producing Bregs have higher mitochondrial mass over other B cells (238). Despite this reliance on OxPhos, IL-10 is under the control of Hif-1α and ERK signaling pathways (238).Bregs have demonstrated the ability to reduce hypoxia-induced pathology caused by a Tfh/Tfr imbalance. Bregs were seen to control hypoxia derived pulmonary hypertension as mice that had exogenous Bregs transferred into them presented with amplified Tfr numbers, preventing the aberrant expansion of Tfh cells (239). Bregs and their cytokines, such as IL-10, have been demonstrated to impact immunological responses to prevent various pathologies (239–241).

Plasma cells are a main contributor of B cell derived IL-10 and IL-35 (242). Without IL-35 coming from plasma cells, there is a significant delay in recovery from autoimmune disorders like experimental autoimmune encephalomyelitis (242). Inversely, IL-35 produced by B cells increases susceptibility to infection (242). When IL-35 is depleted within B cells there is an upregulation of interferon signaling, phagocytosis, and expanded activated T cell populations (242). Intestinal mucosa IgG^+^ plasma cells principally use OxPhos for energy (243). The authors of this study suggest that this consumption of oxygen can contribute to mucosal hypoxia and exacerbate ulcerative colitis (243). Interestingly, upregulation of CD11b on B cells in a Hif-1α dependent manner was found to be important for Breg function (244). It is worth noting that Hif-1α can be stabilized in hypoxia-independent manners. One such manner is activation of the PKC pathway which upregulates transcription of Hif-1α mRNA (245). This has been suggested to outpace proteasomal degradation facilitating HRE transcription (245). Other oxygen-independent mechanisms of Hif regulation have been reviewed by the Kurelac group (34). It is currently unclear if these mechanisms are influential in Breg physiology. As there are no definitive separation criteria between Bregs and other B cell subsets, it is unclear at what point there is a commitment to this lineage. Do B cells have a level of plasticity similar to T cells? There is much we do not understand concerning this cell type, and the role hypoxia might play on controlling Breg cell fate. Current data suggests that Bregs may be under the control of hypoxic signaling to induce their immunosuppressive functions, and this may be tied to where they often reside (e.g. gut mucosa).

## Conclusions

The cellular response to hypoxia is one of the physiologically oldest and most phylogenetically conserved immunoregulatory pathways. There is a growing body of research characterizing hypoxia as a normal physiological parameter instead of solely a pathological state. Movement of lymphocytes through various oxygen tensions in bone marrow and thymus have been demonstrated to be vital for proper B and T cell development. Interestingly, hypoxic microenvironments that develop during the inflammatory response has also been shown to limit ongoing immune responses and protect tissues from collateral damage due to excessive inflammation. Understanding this complex relationship between what factors of hypoxia improve immunological responses and what hypoxia derived factors control immunological outgrowth could inform successful immunotherapies and vaccine design.

The hypoxia-adenosinergic pathway has been shown to regulate both cell-mediated and humoral immunity. While manipulation of this signaling pathway is an established approach to promote effective anti-tumor T cell responses in improving cancer immunotherapies (14, 205, 206, 208, 218), the role for this pathway in regulating B cell responses is still being explored. The discovery that the GC develops a hypoxic microenvironment and is under the control of adenosine receptors may lead to novel hypoxia-adenosinergic targeting therapies that can enhance vaccine responses. These improvements could lead to increased vaccine durability, lowering the requisite number of vaccinations to elicit immunity, or enabling development of broadly protective anti-pathogen antibodies to difficult targets such as HIV or influenza.
